# 1-Butyl-3-ethyl-1*H*-benzimidazol-3-ium tetra­fluoro­borate

**DOI:** 10.1107/S1600536812037476

**Published:** 2012-09-05

**Authors:** Denise M. Junge, Derek R. Scadova, James A. Golen, Jerry P. Jasinski

**Affiliations:** a Department of Chemistry, Keene State College, 229 Main Street, Keene, NH 03435-2001, USA

## Abstract

In the title salt, C_13_H_19_N_2_^+^·BF_4_^−^, an ionic liquid, the butyl and ethyl substituents bonded to the N atoms of the imidazole ring [r.m.s. deviation = 0.019 (1) Å] adopt equatorial positions. The crystal structure exhibits slipped π–π inter­actions between the imidazole and benzene rings of neighbouring mol­ecules [centroid–centroid distance = 3.529 (2) Å]. In the tetra­fluoro­borate anion, the B and F atoms are disordered over two sets of sites with site-occupancy factors of 0.813 (7) and 0.187 (7).

## Related literature

For properties of ionic liquids, see: Zhao & Malhotra (2002 ▶) For imidazolium-based ionic liquids, see: Welton (1999 ▶); Hallett & Welton (2011 ▶); Costache *et al.* (2007 ▶); Chen *et al.* (2008 ▶). For the synthesis of ionic liquid compounds, see: Dupont *et al.* (2004 ▶); Huang *et al.* (2004 ▶). For standard bond lengths, see Allen *et al.* (1987 ▶).
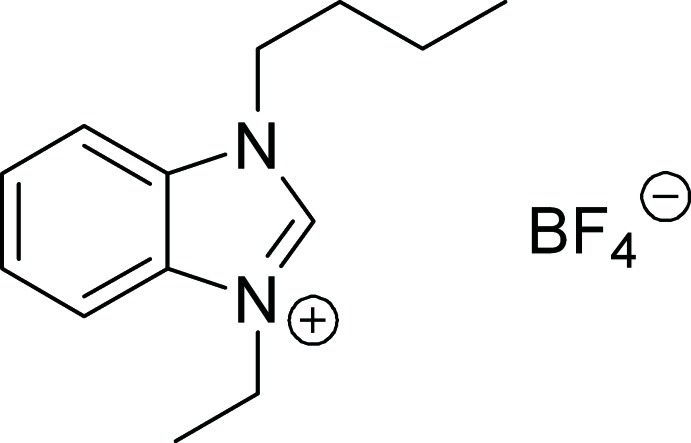


## Experimental

### 

#### Crystal data

C_13_H_19_N_2_^+^·BF_4_^−^
*M* *_r_* = 290.11
Monoclinic, 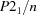

*a* = 11.0043 (13) Å
*b* = 12.0372 (9) Å
*c* = 11.3693 (10) Å
β = 99.312 (9)°
*V* = 1486.1 (2) Å^3^
*Z* = 4
Cu *K*α radiation
μ = 0.96 mm^−1^
*T* = 173 K
0.29 × 0.24 × 0.20 mm

#### Data collection

Oxford Diffraction Xcalibur Eos Gemini diffractometer
Absorption correction: multi-scan (*CrysAlis PRO*; Oxford Diffraction, 2010 ▶) *T*_min_ = 0.769, *T*_max_ = 0.831
9159 measured reflections
2860 independent reflections
2655 reflections with *I* > 2σ(*I*)
*R*_int_ = 0.024

#### Refinement

*R*[*F* ^2^ > 2σ(*F* ^2^)] = 0.048
*wR*(*F* ^2^) = 0.137
*S* = 1.05
2860 reflections
229 parameters
68 restraints
H-atom parameters constrained
Δρ_max_ = 0.46 e Å^−3^
Δρ_min_ = −0.25 e Å^−3^

### 

Data collection: *CrysAlis PRO* (Oxford Diffraction, 2010 ▶); cell refinement: *CrysAlis PRO*; data reduction: *CrysAlis RED* (Oxford Diffraction, 2010 ▶); program(s) used to solve structure: *SHELXS97* (Sheldrick, 2008 ▶); program(s) used to refine structure: *SHELXL97* (Sheldrick, 2008 ▶); molecular graphics: *SHELXTL* (Sheldrick, 2008 ▶) and *Mercury* (Macrae *et al.*, 2006 ▶); software used to prepare material for publication: *SHELXTL*.

## Supplementary Material

Crystal structure: contains datablock(s) global, I. DOI: 10.1107/S1600536812037476/lx2260sup1.cif

Structure factors: contains datablock(s) I. DOI: 10.1107/S1600536812037476/lx2260Isup2.hkl

Supplementary material file. DOI: 10.1107/S1600536812037476/lx2260Isup3.cml

Additional supplementary materials:  crystallographic information; 3D view; checkCIF report

## Figures and Tables

**Fig. 1.**
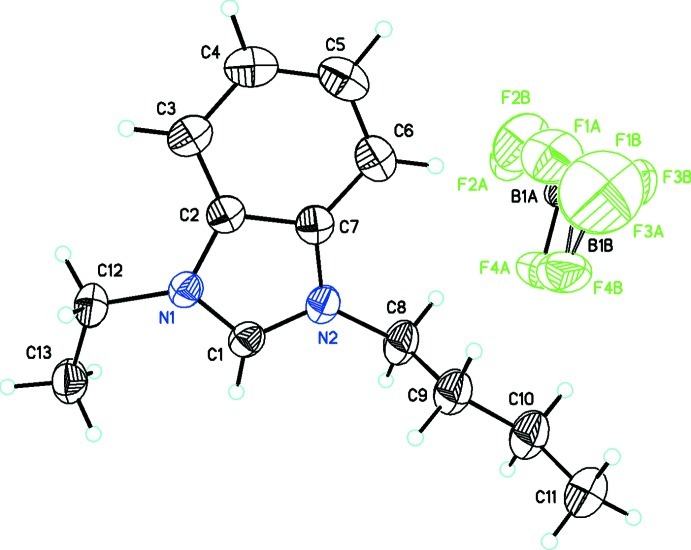
The molecular structure of the title compound with the atom numbering scheme. Displacement ellipsoids are drawn at the 50% probability level. H atoms are presented as small spheres of arbitrary radius. The B and F atoms of the tetrafluoroborate group are disordered over two positions with refined site-occupancy factors of 0.813 (7) (part A) and 0.187 (7) (part B).

**Fig. 2.**
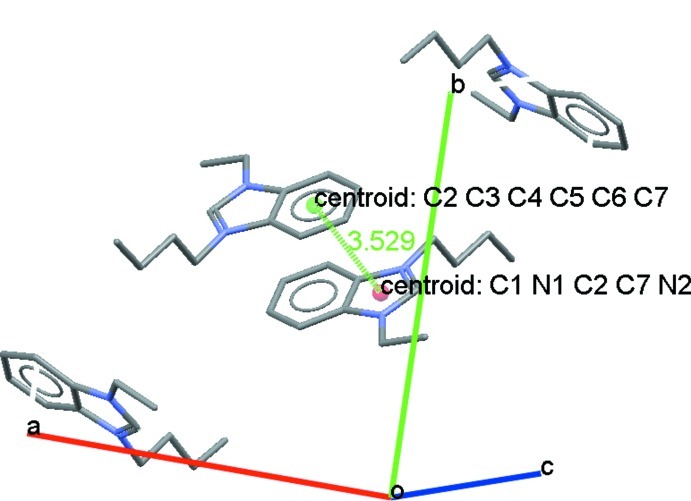
A view of the π–π interactions (dotted lines) in the crystal structure of the title compound. (Cg1 and Cg2 are the centroids of the N1/C1/N2/C7/C2 imidazole ring and the C2–C7 benzene ring, respectively; Symmetry code: 1-x, 1-y, 1-z). Disordered tetrafluoroborate group and all H atoms were omitted for clarity.

## supplementary crystallographic information

### Comment

Due to their unique properties, ionic liquids have emerged as environmentally friendly alternatives for volatile organic solvents (Zhao *et al.*, 2002). In particular, imidazolium-based ionic liquids have been used as solvents and catalysts for a wide variety of chemical processes (Welton, 1999; Hallett *et al.*, 2011). Benzimidazole can be viewed as a homologue of imidazole and, therefore, similar properties and applications as seen with the imidazolium-based ionic liquids is expected (Costache *et al.*, 2007; Chen *et al.*, 2008). In continuation of our work with ionic liquids, we report herein the crystal structure of the title compound.

In the title molecule (Fig. 1), imidazole ring is essentially planar, with a mean deviation of 0.019 (1) Å from the least-squares plane defined by the five constituent atoms. In the tetrafluoroborate group, the B and F atoms are disordered over two positions with site-occupancy factors, from refinement of 0.813 (7) (part A) and 0.187 (7) (part B). The butyl and ethyl substituents bonded to the nitrogen atoms with the mean plane of the imidazole ring adopt equatorial positions. Bond lengths are in normal ranges (Allen *et al.*, 1987).

The crystal packing (Fig. 2) exhibits slipped π–π intermolecular stacking interactions between the imidazole and benzene rings of neighbouring molecules, with a Cg1-Cg2 distance of 3.5300 (11) Å and an interplanar distance of 3.529 (3)Å resulting in a slippage of 3.11 (2)Å (Fig. 2) (Cg1 and Cg2 are the centroids of the N1/C1/N2/C7/C2 imidazole ring and the C2–C7 benzene ring, respectively). In the crystal structure the disordered C—H···F interactions were ignored.

### Experimental

1-butylbenzimidazole (1.001 g, 5.74 mmol) and ethyl bromide (471µ*L*, 6.31mmol) were combined in a sample vial equipped with a stir bar. The mixture washeated at 80 °C in an oil bath for 24 h. Once cooled, 3 ml of acetonitrile was added to dissolve the mixture and toluene was then added drop wise until the mixture turned cloudy (8–10 ml). The mixture was then cooled, filtered, and dried under vacuum to yield 1-butyl-3-ethyl-1*H*-benzimidazol-3-iumbromide. 1-Butyl-3-ethyl-1*H*-benzimidazol-3-ium bromide (250 mg, 0.883mmol), sodium tetrafluoroborate (97 mg, 0.883 mmol), and distilled water (5ml) were combined in a 25 ml round-bottom flask and allowed to stir at roomtemperature for 24 h. The reaction mixture was then extracted with dichloromethane (4 *x* 5 ml) and dried over Na_2_SO_4_. The dichloromethane was removed solvent by rotary evaporation, and dried under vacuum to yield the title product (m.p.: 354 - 356 K).

### Refinement

The B and F atoms in the tetrafluoroborate anion are disordered over two sets of site with an occupancy ratio: 0.813 (7):0.187 (7) and with all B—F distances fixed at 1.36 (2)Å with ISOR (s = 0.01) constraints applied. In the cation, all of the H atoms were placed in their calculated positions and then refined using the riding model with C—H lengths of 0.95 Å (CH), 0.99 Å (CH_2_) or 0.98 Å (CH_3_). The isotropic displacement parameters for these atoms were set to 1.18 to 1.20 (CH), 1.20 (CH_2_or 1.50 (CH_3_) times *U*_eq_ of the parent atom.

### Crystal data

**Table:**

C_13_H_19_N_2_^+^·BF_4_^−^	*F*(000) = 608
*M**_r_* = 290.11	*D*_x_ = 1.297 Mg m^−^^3^
Monoclinic, *P*2_1_/*n*	Cu *K*α radiation, λ = 1.54178 Å
Hall symbol: -P 2yn	Cell parameters from 4678 reflections
*a* = 11.0043 (13) Å	θ = 3.7–71.2°
*b* = 12.0372 (9) Å	µ = 0.96 mm^−^^1^
*c* = 11.3693 (10) Å	*T* = 173 K
β = 99.312 (9)°	Block, colorless
*V* = 1486.1 (2) Å^3^	0.29 × 0.24 × 0.20 mm
*Z* = 4	

### Data collection

**Table:**

Oxford Diffraction Xcalibur Eos Gemini diffractometer	2860 independent reflections
Radiation source: Enhance (Cu) X-ray Source	2655 reflections with *I* > 2σ(*I*)
Graphite monochromator	*R*_int_ = 0.024
Detector resolution: 16.1500 pixels mm^-1^	θ_max_ = 71.3°, θ_min_ = 5.2°
ω scans	*h* = −13→13
Absorption correction: multi-scan (*CrysAlis PRO*; Oxford Diffraction, 2010)	*k* = −14→10
*T*_min_ = 0.769, *T*_max_ = 0.831	*l* = −13→13
9159 measured reflections	

### Refinement

**Table:**

Refinement on *F*^2^	Primary atom site location: structure-invariant direct methods
Least-squares matrix: full	Secondary atom site location: difference Fourier map
*R*[*F*^2^ > 2σ(*F*^2^)] = 0.048	Hydrogen site location: inferred from neighbouring sites
*wR*(*F*^2^) = 0.137	H-atom parameters constrained
*S* = 1.05	*w* = 1/[σ^2^(*F*_o_^2^) + (0.0742*P*)^2^ + 0.572*P*] where *P* = (*F*_o_^2^ + 2*F*_c_^2^)/3
2860 reflections	(Δ/σ)_max_ < 0.001
229 parameters	Δρ_max_ = 0.46 e Å^−^^3^
68 restraints	Δρ_min_ = −0.25 e Å^−^^3^

### Special details

**Table:**

Geometry. All esds (except the esd in the dihedral angle between two l.s. planes) are estimated using the full covariance matrix. The cell esds are taken into account individually in the estimation of esds in distances, angles and torsion angles; correlations between esds in cell parameters are only used when they are defined by crystal symmetry. An approximate (isotropic) treatment of cell esds is used for estimating esds involving l.s. planes.
Refinement. Refinement of *F*^2^ against ALL reflections. The weighted *R*-factor *wR* and goodness of fit *S* are based on *F*^2^, conventional *R*-factors *R* are based on *F*, with *F* set to zero for negative *F*^2^. The threshold expression of *F*^2^ > σ(*F*^2^) is used only for calculating *R*-factors(gt) *etc*. and is not relevant to the choice of reflections for refinement. *R*-factors based on *F*^2^ are statistically about twice as large as those based on *F*, and *R*- factors based on ALL data will be even larger.

### Fractional atomic coordinates and isotropic or equivalent isotropic displacement parameters (Å^2^)

**Table:**

	*x*	*y*	*z*	*U*_iso_*/*U*_eq_	Occ. (<1)
N1	0.71813 (12)	0.62371 (10)	0.52811 (11)	0.0306 (3)	
N2	0.69467 (12)	0.52167 (11)	0.36726 (11)	0.0338 (3)	
C1	0.77356 (14)	0.55764 (12)	0.45961 (13)	0.0323 (3)	
H1A	0.8585	0.5388	0.4749	0.039*	
C2	0.59463 (14)	0.63191 (12)	0.47691 (13)	0.0311 (3)	
C3	0.49671 (16)	0.68773 (14)	0.51404 (15)	0.0378 (4)	
H3A	0.5075	0.7331	0.5833	0.045*	
C4	0.38307 (16)	0.67323 (15)	0.44437 (17)	0.0434 (4)	
H4A	0.3134	0.7094	0.4664	0.052*	
C5	0.36742 (16)	0.60653 (15)	0.34185 (17)	0.0450 (4)	
H5A	0.2873	0.5982	0.2970	0.054*	
C6	0.46499 (16)	0.55249 (14)	0.30407 (15)	0.0408 (4)	
H6A	0.4544	0.5082	0.2340	0.049*	
C7	0.57954 (15)	0.56672 (12)	0.37449 (14)	0.0329 (3)	
C8	0.72604 (18)	0.44867 (14)	0.27240 (15)	0.0428 (4)	
H8A	0.6572	0.3964	0.2475	0.051*	
H8B	0.7998	0.4044	0.3043	0.051*	
C9	0.75105 (18)	0.51369 (15)	0.16499 (15)	0.0439 (4)	
H9A	0.8164	0.5692	0.1906	0.053*	
H9B	0.6756	0.5542	0.1298	0.053*	
C10	0.7910 (2)	0.43834 (17)	0.07123 (17)	0.0509 (5)	
H10A	0.8640	0.3952	0.1079	0.061*	
H10B	0.7240	0.3850	0.0434	0.061*	
C11	0.8223 (2)	0.50152 (19)	−0.03470 (17)	0.0549 (5)	
H11A	0.8472	0.4491	−0.0923	0.082*	
H11B	0.8901	0.5531	−0.0081	0.082*	
H11C	0.7500	0.5433	−0.0724	0.082*	
C12	0.77365 (16)	0.67731 (15)	0.64023 (14)	0.0390 (4)	
H12A	0.7221	0.6616	0.7020	0.047*	
H12B	0.7744	0.7587	0.6282	0.047*	
C13	0.90314 (17)	0.63838 (17)	0.68417 (16)	0.0476 (5)	
H13A	0.9340	0.6737	0.7609	0.071*	
H13B	0.9561	0.6586	0.6261	0.071*	
H13C	0.9035	0.5575	0.6941	0.071*	
B1A	0.4707 (5)	0.2091 (4)	0.1379 (6)	0.0362 (11)	0.813 (7)
F1A	0.4128 (2)	0.30962 (17)	0.1306 (3)	0.0838 (9)	0.813 (7)
F2A	0.4454 (2)	0.1576 (2)	0.23845 (16)	0.0761 (8)	0.813 (7)
F3A	0.4286 (6)	0.1443 (5)	0.0392 (4)	0.0713 (13)	0.813 (7)
F4A	0.5969 (3)	0.2217 (2)	0.1492 (3)	0.0582 (7)	0.813 (7)
B1B	0.472 (2)	0.2157 (18)	0.112 (2)	0.040 (7)	0.187 (7)
F1B	0.4416 (19)	0.3070 (16)	0.052 (2)	0.171 (7)	0.187 (7)
F2B	0.4146 (12)	0.2333 (16)	0.2030 (13)	0.118 (5)	0.187 (7)
F3B	0.443 (3)	0.122 (2)	0.061 (2)	0.077 (6)	0.187 (7)
F4B	0.5827 (15)	0.2487 (14)	0.1055 (15)	0.081 (4)	0.187 (7)

### Atomic displacement parameters (Å^2^)

**Table:**

	*U*^11^	*U*^22^	*U*^33^	*U*^12^	*U*^13^	*U*^23^
N1	0.0337 (7)	0.0301 (6)	0.0287 (6)	−0.0005 (5)	0.0076 (5)	0.0000 (5)
N2	0.0407 (7)	0.0313 (7)	0.0305 (6)	−0.0021 (5)	0.0095 (5)	−0.0023 (5)
C1	0.0353 (8)	0.0311 (7)	0.0320 (8)	−0.0006 (6)	0.0097 (6)	0.0010 (6)
C2	0.0354 (8)	0.0278 (7)	0.0307 (7)	−0.0009 (6)	0.0072 (6)	0.0064 (6)
C3	0.0429 (9)	0.0336 (8)	0.0391 (8)	0.0045 (7)	0.0134 (7)	0.0079 (6)
C4	0.0383 (9)	0.0402 (9)	0.0533 (10)	0.0064 (7)	0.0118 (7)	0.0178 (8)
C5	0.0376 (9)	0.0426 (9)	0.0515 (10)	−0.0040 (7)	−0.0026 (7)	0.0186 (8)
C6	0.0466 (9)	0.0359 (8)	0.0375 (8)	−0.0073 (7)	0.0000 (7)	0.0076 (7)
C7	0.0383 (8)	0.0288 (7)	0.0318 (7)	−0.0028 (6)	0.0068 (6)	0.0055 (6)
C8	0.0571 (11)	0.0363 (8)	0.0367 (9)	−0.0009 (7)	0.0122 (8)	−0.0094 (7)
C9	0.0543 (10)	0.0407 (9)	0.0379 (9)	−0.0013 (8)	0.0112 (8)	−0.0060 (7)
C10	0.0673 (12)	0.0467 (10)	0.0409 (10)	−0.0011 (9)	0.0154 (9)	−0.0084 (8)
C11	0.0645 (13)	0.0609 (12)	0.0415 (10)	0.0026 (10)	0.0156 (9)	−0.0045 (9)
C12	0.0463 (9)	0.0391 (8)	0.0309 (8)	0.0022 (7)	0.0042 (7)	−0.0065 (6)
C13	0.0464 (10)	0.0588 (11)	0.0357 (9)	0.0040 (8)	0.0005 (7)	−0.0104 (8)
B1A	0.0314 (19)	0.039 (2)	0.040 (2)	−0.0010 (13)	0.0120 (14)	−0.0148 (14)
F1A	0.0794 (13)	0.0589 (11)	0.113 (2)	0.0298 (9)	0.0161 (12)	−0.0073 (11)
F2A	0.0966 (15)	0.0817 (15)	0.0552 (10)	−0.0204 (11)	0.0273 (9)	−0.0024 (9)
F3A	0.0738 (19)	0.088 (3)	0.0556 (13)	−0.033 (2)	0.0194 (11)	−0.0336 (17)
F4A	0.0376 (9)	0.0520 (11)	0.0852 (17)	−0.0042 (8)	0.0103 (10)	−0.0151 (11)
B1B	0.036 (9)	0.044 (9)	0.041 (10)	−0.005 (6)	0.006 (6)	−0.008 (6)
F1B	0.183 (11)	0.153 (10)	0.175 (11)	0.065 (8)	0.025 (8)	0.047 (8)
F2B	0.112 (7)	0.144 (11)	0.103 (7)	0.007 (7)	0.036 (6)	−0.032 (8)
F3B	0.075 (8)	0.056 (6)	0.111 (11)	−0.025 (5)	0.044 (8)	−0.024 (6)
F4B	0.060 (6)	0.085 (8)	0.104 (9)	−0.025 (6)	0.031 (6)	−0.031 (6)

### Geometric parameters (Å, º)

**Table:**

N1—C1	1.3283 (19)	C9—H9B	0.9900
N1—C2	1.393 (2)	C10—C11	1.511 (3)
N1—C12	1.471 (2)	C10—H10A	0.9900
N2—C1	1.322 (2)	C10—H10B	0.9900
N2—C7	1.393 (2)	C11—H11A	0.9800
N2—C8	1.475 (2)	C11—H11B	0.9800
C1—H1A	0.9500	C11—H11C	0.9800
C2—C7	1.392 (2)	C12—C13	1.506 (2)
C2—C3	1.392 (2)	C12—H12A	0.9900
C3—C4	1.379 (3)	C12—H12B	0.9900
C3—H3A	0.9500	C13—H13A	0.9800
C4—C5	1.403 (3)	C13—H13B	0.9800
C4—H4A	0.9500	C13—H13C	0.9800
C5—C6	1.382 (3)	B1A—F1A	1.364 (5)
C5—H5A	0.9500	B1A—F2A	1.368 (7)
C6—C7	1.390 (2)	B1A—F4A	1.382 (5)
C6—H6A	0.9500	B1A—F3A	1.383 (6)
C8—C9	1.513 (2)	B1B—F3B	1.288 (19)
C8—H8A	0.9900	B1B—F4B	1.291 (19)
C8—H8B	0.9900	B1B—F1B	1.307 (19)
C9—C10	1.518 (2)	B1B—F2B	1.315 (18)
C9—H9A	0.9900	F1B—F4B	1.72 (3)
			
C1—N1—C2	107.88 (13)	C11—C10—C9	112.86 (16)
C1—N1—C12	127.24 (14)	C11—C10—H10A	109.0
C2—N1—C12	124.86 (13)	C9—C10—H10A	109.0
C1—N2—C7	108.19 (13)	C11—C10—H10B	109.0
C1—N2—C8	125.07 (14)	C9—C10—H10B	109.0
C7—N2—C8	126.73 (14)	H10A—C10—H10B	107.8
N2—C1—N1	110.90 (14)	C10—C11—H11A	109.5
N2—C1—H1A	124.5	C10—C11—H11B	109.5
N1—C1—H1A	124.5	H11A—C11—H11B	109.5
C7—C2—C3	122.17 (15)	C10—C11—H11C	109.5
C7—C2—N1	106.62 (13)	H11A—C11—H11C	109.5
C3—C2—N1	131.18 (15)	H11B—C11—H11C	109.5
C4—C3—C2	116.06 (16)	N1—C12—C13	112.89 (14)
C4—C3—H3A	122.0	N1—C12—H12A	109.0
C2—C3—H3A	122.0	C13—C12—H12A	109.0
C3—C4—C5	121.86 (16)	N1—C12—H12B	109.0
C3—C4—H4A	119.1	C13—C12—H12B	109.0
C5—C4—H4A	119.1	H12A—C12—H12B	107.8
C6—C5—C4	122.04 (16)	C12—C13—H13A	109.5
C6—C5—H5A	119.0	C12—C13—H13B	109.5
C4—C5—H5A	119.0	H13A—C13—H13B	109.5
C5—C6—C7	116.15 (16)	C12—C13—H13C	109.5
C5—C6—H6A	121.9	H13A—C13—H13C	109.5
C7—C6—H6A	121.9	H13B—C13—H13C	109.5
C6—C7—C2	121.71 (15)	F1A—B1A—F2A	107.2 (4)
C6—C7—N2	131.82 (15)	F1A—B1A—F4A	111.2 (4)
C2—C7—N2	106.41 (13)	F2A—B1A—F4A	108.1 (5)
N2—C8—C9	112.13 (14)	F1A—B1A—F3A	111.0 (5)
N2—C8—H8A	109.2	F2A—B1A—F3A	109.6 (4)
C9—C8—H8A	109.2	F4A—B1A—F3A	109.7 (5)
N2—C8—H8B	109.2	F3B—B1B—F4B	114 (2)
C9—C8—H8B	109.2	F3B—B1B—F1B	119 (2)
H8A—C8—H8B	107.9	F4B—B1B—F1B	83.0 (17)
C8—C9—C10	111.65 (15)	F3B—B1B—F2B	112 (2)
C8—C9—H9A	109.3	F4B—B1B—F2B	125 (2)
C10—C9—H9A	109.3	F1B—B1B—F2B	98.9 (19)
C8—C9—H9B	109.3	B1B—F1B—F4B	48.1 (11)
C10—C9—H9B	109.3	B1B—F4B—F1B	48.9 (11)
H9A—C9—H9B	108.0		
			
C7—N2—C1—N1	0.12 (17)	C3—C2—C7—N2	−178.24 (13)
C8—N2—C1—N1	178.73 (14)	N1—C2—C7—N2	−0.17 (15)
C2—N1—C1—N2	−0.23 (17)	C1—N2—C7—C6	−176.98 (16)
C12—N1—C1—N2	178.14 (14)	C8—N2—C7—C6	4.4 (3)
C1—N1—C2—C7	0.25 (16)	C1—N2—C7—C2	0.04 (16)
C12—N1—C2—C7	−178.17 (14)	C8—N2—C7—C2	−178.54 (14)
C1—N1—C2—C3	178.08 (15)	C1—N2—C8—C9	−95.55 (19)
C12—N1—C2—C3	−0.3 (2)	C7—N2—C8—C9	82.8 (2)
C7—C2—C3—C4	1.0 (2)	N2—C8—C9—C10	176.41 (15)
N1—C2—C3—C4	−176.50 (14)	C8—C9—C10—C11	−177.36 (17)
C2—C3—C4—C5	−0.3 (2)	C1—N1—C12—C13	−7.8 (2)
C3—C4—C5—C6	−0.7 (3)	C2—N1—C12—C13	170.28 (15)
C4—C5—C6—C7	0.9 (2)	F3B—B1B—F1B—F4B	−114 (3)
C5—C6—C7—C2	−0.1 (2)	F2B—B1B—F1B—F4B	125 (2)
C5—C6—C7—N2	176.50 (15)	F3B—B1B—F4B—F1B	118 (3)
C3—C2—C7—C6	−0.8 (2)	F2B—B1B—F4B—F1B	−96 (3)
N1—C2—C7—C6	177.22 (13)		
